# Association of IBD specific treatment and prevalence of pain in the Swiss IBD cohort study

**DOI:** 10.1371/journal.pone.0215738

**Published:** 2019-04-25

**Authors:** Lorenz Bon, Sylvie Scharl, Stephan Vavricka, Gerhard Rogler, Nicolas Fournier, Valerie Pittet, Michael Scharl, Thomas Greuter, Philipp Schreiner, Pascal Frei, Benjamin Misselwitz, Luc Biedermann, Jonas Zeitz, for the Swiss IBD Cohort Study Group

**Affiliations:** 1 Division of Gastroenterology and Hepatology, University Hospital Zurich, University of Zurich, Zurich, Switzerland; 2 Zurich Center for Integrative Human Physiology, University of Zurich, Zurich, Switzerland; 3 Institute of Social and Preventive Medicine, University of Lausanne, Lausanne, Switzerland; 4 Gastroenterology Bethanien, Zurich, Switzerland; 5 Center of Gastroenterology, Clinic Hirslanden, Zurich, Switzerland; Humanitas University, ITALY

## Abstract

**Background:**

Extraintestinal manifestations (EIM) contribute significantly to the burden of disease in inflammatory bowel disease (IBD). Pain is a leading symptom in IBD and could be seen as an EIM itself. Treatment of IBD associated pain is challenging and insufficiently studied. A better knowledge on the association of pain and IBD specific treatment is warranted to improve the management of IBD patients.

**Methods:**

All patients of the Swiss IBD Cohort Study (SIBDCS) (n = 2152) received a questionnaire regarding pain localization, pain character, and the use of IBD specific medication.

**Results:**

1263 completed questionnaires were received. Twenty-one out of 184 patients (10%) receiving anti-TNF treatment compared to 142 out of 678 patients (21%) not receiving anti-TNF medication reported elbow pain (p = 0.002) while 28 out of 198 patients (14%) receiving steroid treatment significantly more often reported elbow pain compared to 59 from 696 patients (8%) not receiving steroids (p = 0.021). Furthermore, we found significantly more female patients under anti-TNF treatment to report knee/ lower leg pain and ankle/ foot pain compared to their male counterparts (36% vs. 20% and 22% vs. 10%, respectively, p = 0.015 for both comparisons). The frequency of knee, lower leg, ankle and foot pain was especially low in male patients under anti-TNF treatment, indicating a high benefit of male patients from anti-TNF therapy regarding EIM.

**Conclusions:**

The frequency of elbow pain was lower in IBD patients treated with anti-TNF but higher in patients treated with steroids.

## Introduction

Pain is a common symptom in patients with inflammatory bowel disease (IBD) [1, 2]. In a recent study we showed that the vast majority of patients (71%) within the Swiss IBD Cohort Study experienced pain during their disease course and that for 52% of the patients pain was a longstanding problem [3]. Abdominal pain can be a direct or indirect consequence of intestinal inflammation; however, extraintestinal manifestations (EIM) of IBD can also cause pain and pain in itself can be seen as an EIM [4–6]. The most common EIM of IBD are arthropathies [5, 7–17]. Also, in our former study we could show that pain has a substantial impact on health-related quality of life (HRQOL) of IBD patients, as the general quality of life was significantly lower in patients suffering from pain compared to those without pain [3]. Such a relationship has also been described in other chronic diseases [18–21].

Treatment of both, IBD and IBD associated pain is challenging. The mainstay of IBD treatment includes systemic immunosuppressive medications, such as corticosteroids, anti-tumor necrosis factor (TNF) antibodies or immunomodulators. Furthermore, the management of an acute flare differs from the strategies for maintenance of remission [22, 23]. Moreover, presence of EIM will also influence the choice of a treatment regime. For instance, anti-TNF therapy is known to be very effective regarding gut inflammation as well as arthropathies/ arthritis.

Furthermore, non-steroidal anti-inflammatory drugs (NSAIDs) can very effectively mediate pain relieve due to their analgetic and also anti-inflammatory effects. However, due to the risk of disease exacerbation and induction of flares their use in IBD is limited [24–30].

Here, we used the well-characterized patient collective of the Swiss IBD Cohort Study (SIBDCS) to study the association of pain and IBD treatment with a focus on anti-TNF treatment.

## Methods

### Ethics consideration

Ethics approval was obtained from the regional Swiss Ethics Committees in which cohort participants were enrolled (Commission d’éthique du Canton de Vaud, Lausanne, Switzerland/Protocol no. 33/06). Written, informed consent was obtained from each patient included in the study. The study protocol conforms to the ethical guidelines of the 1975 Declaration of Helsinki as reflected in a priori approval by the institution's human research committee.

### Study design

Patients of the nationwide SIBDCS have been prospectively included since 2006 with a yearly follow-up. The cohort goals und methodology of SIBDC have been described elsewhere[31].

A questionnaire addressing various aspects of pain including pain duration localization and frequency was mailed to 2152 SIBDC patients, representing the entire cohort. The questionnaire also inquired about the use of pain specific medication in detail. Our questionnaire contained several questions from a validated German pain questionnaire [32]. The questionnaire was used in a German and in a French version. Further details of the questionnaires including the fully originally used French and German versions are described elsewhere [3]. Basic epidemiological and clinical data including the use of IBD specific therapy was retrieved from the SIBDCS databases. All data are stored in Microsoft Access (Microsoft Corporation) databases.

### Statistical analysis

Descriptive statistical analyses were performed: Categorical variables were summarized as frequencies and percentages, whereas quantitative variables as median and range. To assess differences in categorical data distribution between groups of different sizes, Fisher’s exact test was used.

The statistical analysis was performed using GraphPad Prism 7 for MacOS. A p-value of <0.05 was considered statistically significant.

## Results

### Patient’s characteristics

The patients’ characteristics shown in Table 1 have been described previously [3]. In brief: 1263 out of 2152 patients completed the questionnaires (response rate 59%). 599 out of 1263 patients were male (47%) and 664 female (53%). The median age was 47 years. Extraintestinal manifestations (EIM) of IBD were present in 699 patients (55%). The median IBD disease duration was 15 years (mean: 15 years, range: 0–57 years). The vast majority of patients (894/ 1263, 71%) reported the experience of pain in general during the course of the disease. Table 2 shows the frequency of IBD specific treatment.

**Table 1:** Patient characteristics.

Patient characteristics		Number of patients (%)
**Gender**	**Female**	664 (53)
	**Male**	599 (47)
**Diagnosis**	**CD**	679 (54)
	**UC**	556 (44)
	**IC**	28 (2)
	**Sum**	1263 (100)
**Pain**	**Yes**	894 (71)
	**No**	369 (29)
**EIM**	**Yes**	699 (55)
	**No**	564 (45)
**Disease duration (Years)**	**Average**	15
	**Min-Max**	0–57

**Table 2:** IBD specific treatment.

IBD treatment	Number of patients (%) with pain without pain	
**Anti-TNF**	216 (24.2)	100 (27.1)
**Steroids**	198 (22.1)	73 (19.8)
**5-aminosalicylic acid (5-ASA)**	334 (37.4)	136 (36.9)
**Antibiotics**	11 (1.2)	6 (1.6)
**Calcineurin-Inhibitors**	12 (1.3)	5 (1.4)
**Immunomodulators**	316 (35.3)	125(33.9)

### Association between IBD specific treatment and pain localization

When comparing the use of IBD specific medication and ten different pain localizations, we found several significant differences. Regarding elbow pain, only 21 patients (10%) receiving anti-TNF treatment compared to 142 patients (21%) not receiving anti-TNF were affected (p = 0.002). Other pain localizations did not reveal significant differences regarding anti-TNF treatment (Table 3).

**Table 3:** Pain localization.

	Anti-TNF	No anti-TNF	
Pain localization	N (%)	N (%)	p-value
**Head**	56 (26)	147 (21.7)	0.193
**Neck**	28 (13)	95 (14)	0.735
**Finger/hand**	53 (24.5)	142 (20.9)	0.297
**Elbow**	21 (9.7)	142 (20.9)	**0.002**
**Shoulder**	44 (20.4)	138 (20.4)	>0.999
**Back**	77 (35.6)	236 (34.8)	0.869
**Hip/thigh**	52 (24)	162 (23.9)	>0.999
**Knee/lower leg**	61 (28.2)	181 (26.7)	0.660
**Ankle/foot**	35 (16.2)	109 (16.1)	>0.999
**Abdomen**	105 (48.6)	375 (55.3)	0.099

Comparing other IBD specific therapy and the different pain localization, we found patients not receiving steroid treatment significantly less often to be suffering from elbow pain compared to patients receiving steroids (8% vs. 14%; p = 0.021). For the evaluation of other pain localizations and other IBD specific therapy (5-ASA, calcineurin-inhibitors, immunomodulators) no significant differences were observed (S1–S5 Tables).

### Association between IBD specific treatment and duration of pain

Duration of pain did not differ between patients on anti-TNF treatment versus those not on anti-TNF treatment (Table 4). The duration of pain was also not influenced by other IBD specific medications (Steroids, 5-ASA, Antibiotics, Calcineurin-inhibitors, Immunomudulators) also, no significant differences were observed (S6–S10 Tables).

**Table 4:** Duration of pain.

	Anti-TNF	No anti-TNF	
Pain peroid	N (%)	N (%)	p-value
**<1 month**	6 (2.7)	9 (1.3)	0.218
**1 month-½ year**	20 (9.3)	37 (5.5)	0.054
**½ year-1 year**	13 (6)	46 (6.8)	0.608
**1–2 years**	18 (8.3)	61 (9)	0.890
**2–5 years**	51 (23.6)	164 (24.2)	0.927
**>5 years**	108 (50)	361 (53.2)	0.434

### Association between IBD specific treatment and frequency of pain

The frequency of pain in patients with and without anti-TNF treatment did not significantly differ (Table 5). When comparing the pain frequencies of patients taking other IBD specific medications (steroids, 5-ASA, antibiotics, calcineurin-inhibitors, immunomodulators), also no significant differences were observed (S11–S15 Tables).

**Table 5:** Frequency of pain.

	Anti-TNF	No anti-TNF	
Pain Frequency	N (%)	N (%)	p-value
**Several times daily**	49 (28.2)	115 (22)	0.099
**1x/day**	11 (6.3)	34 (6.5)	>0.999
**Several times per week**	34 (19.5)	100 (19.1)	0.911
**1x/week**	11 (6.3)	26 (5)	0.557
**Several times per month**	28 (16.1)	102 (19.5)	0.369
**1x/month**	12 (6.9)	55 (10.5)	0.182
**<1x/month**	29 (16.7)	91 (17.4)	0.907

### Association between IBD specific treatment and pain character

Further, there was no association between the pain character and the use of IBD specific medication. 36 patients (20%) on anti-TNF treatment described their pain to be constant with slight fluctuations compared to 115 patients (19%) without anti-TNF treatment (p>0.999). Constant pain with strong fluctuations was reported by 15 patients (8%) using anti-TNF treatment and by 64 patients (11%) not receiving anti-TNF treatment (p = 0.331). 115 patients (61%) with TNF treatment and 349 (58%) without anti-TNF treatment experienced pain attacks with pain free intervals (p = 0.554). Pain attacks with constant pain were reported by 30 patients (16%) receiving anti-TNF treatment compared to 75 (12%) not receiving anti-TNF (p = 0.268). When comparing the pain character of patients receiving other IBD specific medication (steroids, 5-ASA, antibiotics, calcineurin-inhibitors, immunomodulators), no significant differences across treatment groups were seen (S16–S21 Tables).

### Association between IBD specific treatment and duration of pain attacks

Moreover, the duration of pain attacks was not influenced by IBD specific medication (Table 6), neither with regards to anti-TNF nor other agents to treat IBD, including steroids, 5-ASA, Antibiotics, Calcineurin-Inhibitors, Immunmodulators (S22–S26 Tables).

**Table 6:** Duration of pain attacks.

	Anti-TNF	No anti-TNF	
Duration of pain attacks	N (%)	N (%)	p-value
**Seconds**	26 (14.9)	61 (11.9)	0.295
**Minutes**	56 (32)	158 (30.8)	0.777
**Hours**	55 (31.4)	175 (34)	0.577
**<3 days**	21 (12)	67 (13)	0.793
**>5 days**	17 (9.7)	53 (10.3)	0.885

### Comparison of pain localization of male and female patients with and without anti-TNF therapy

**Fig 1:**
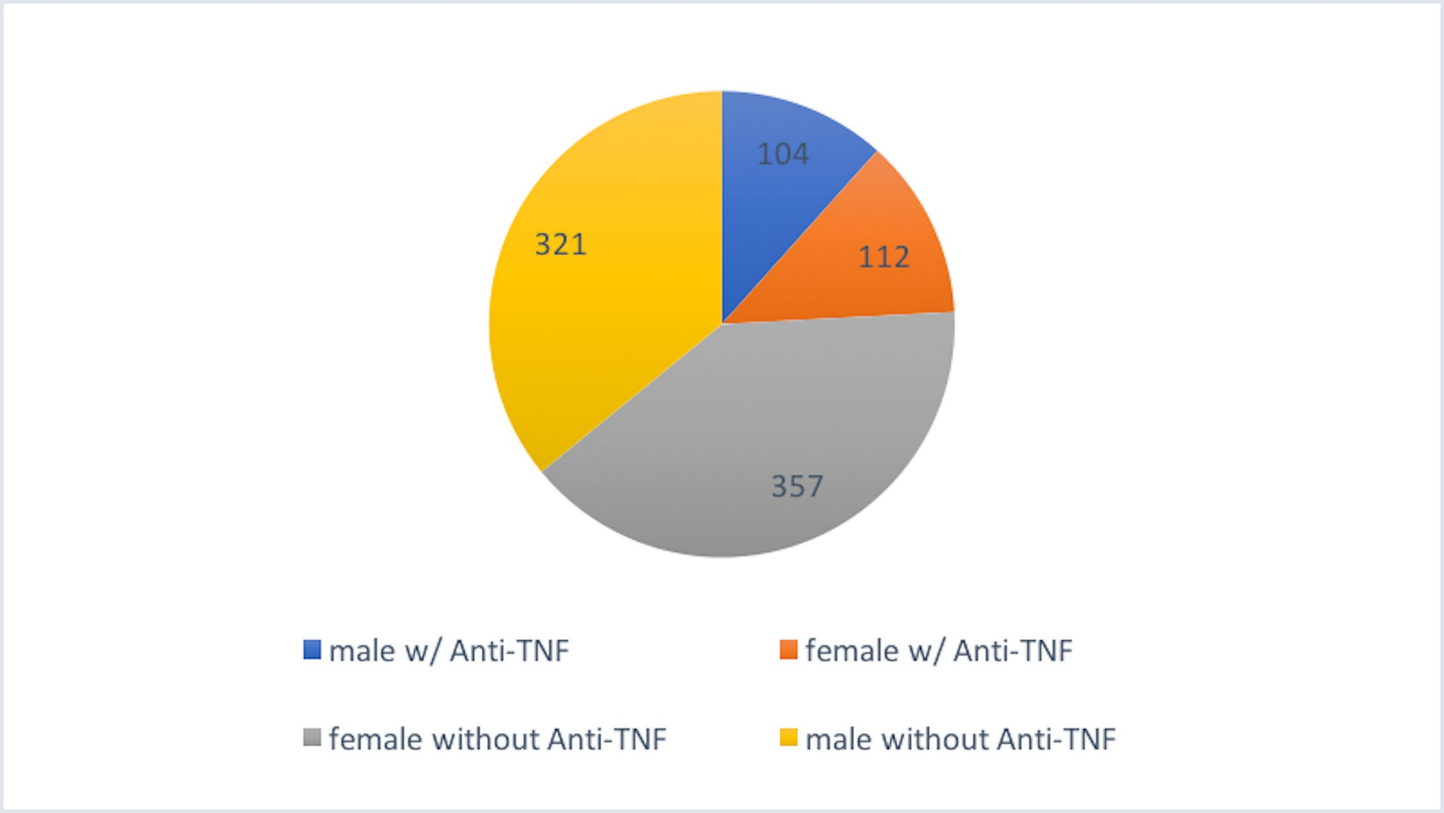
Male and female patients with and without Anti-TNF.

From a total of 894 patients, a similar fraction of male and female patients (24% for both) received anti-TNF therapy (Fig 1).

When comparing the pain localizations of male and female patients receiving anti-TNF treatment significantly fewer male patients with anti-TNF treatment suffered from knee/ lower leg pain compared to female patients receiving anti-TNF therapy (20% vs. 36%; p = 0.015). Also, significantly fewer male patients receiving anti-TNF treatment reported ankle/ foot pain compared to female patients with anti-TNF treatment (10% vs. 22%; p = 0.015). For the other pain localizations, no differences regarding gender were seen (Table 7).

**Table 7:** Pain localization of male vs. female patients with anti-TNF.

	Anti-TNF/ male	Anti-TNF/ female	
Pain localization	N (%)	N (%)	p-value
**Back**	32 (30.8)	45 (40.2)	0.158
**Knee/lower leg**	21 (20.2)	40 (35.7)	**0.015**
**Elbow**	11 (10.6)	10 (8.9)	0.819
**Hip/thigh**	22 (21.2)	30 (26.8)	0.344
**Finger/hand**	21 (20.2)	32 (28.6)	0.158
**Ankle/foot**	10 (9.6)	25 (22.3)	**0.015**

We did not observe any differences in pain localizations in patients with versus without anti-TNF-therapy, neither in male nor female patients (Tables 8 and 9).

**Table 8:** Pain localization of male patients with vs. without anti-TNF.

	Anti-TNF/ male	No anti-TNF/ male	
Pain localization	N (%)	N (%)	p-value
**Back**	32 (30.8)	122 (38)	0.198
**Knee/lower leg**	21 (20.2)	89 (27.7)	0.156
**Elbow**	11 (10.6)	28 (8.7)	0.561
**Hip/thigh**	22 (21.2)	73 (22.7)	0.787
**Finger/hand**	21 (20.2)	69 (21.5)	0.890
**Ankle/foot**	10 (9.6)	51 (15.9)	0.146

**Table 9:** Pain localization of female patients with vs. without anti-TNF.

	anti-TNF/female	No anti-TNF/female	
Pain Localisation	N (%)	N (%)	p-value
**Back**	45 (40.2)	114 (31.9)	0.110
**Knee/lower leg**	40 (35.7)	92 (25.8)	0.053
**Elbow**	10 (8.9)	38 (10.6)	0.721
**Hip/thigh**	30 (26.8)	89 (24.9)	0.709
**Finger/hand**	32 (28.6)	73 (20.4)	0.090
**Ankle/foot**	25 (22.3)	58 (16.2)	0.156

## Discussion

In our study population 5-aminosalycylicacid (5-ASA) (37%) was the most frequently used IBD specific medication, followed by immunomodulators (35%) and anti-TNF antibodies (24%). As for anti-TNF, Vavricka et al. showed that in more than 40% of the cases, this therapy regime is initiated to treat EIM rather than bowel inflammation and over 70% showed a clinical response of EIM to anti-TNF therapy [6]. Our study supports these findings: we could show that significantly less patients on anti-TNF reported elbow pain compared to patients not on anti-TNF. Of note, significantly more patients on steroid treatment reported elbow pain. Refrences to support these findings are lacking.

Regarding gender specific differences in treatment of EIM/ pain in IBD patients, data is not consistent. Concerning IBD treatment, Lopetusa et al. found no general influence of the gender on the therapy of ulcerative colitis (UC) with anti-TNF (infliximab) [33]. However, female patients with steroid-refractory UC and successive anti-TNF treatment showed an increased 1-year remission rate and a cumulative non-colectomy rate. In contradiction, Lopetusa et al. found a lower rate of response to treatment and of disease remission in female patients under TNF inhibitors with axial spondyloarthritis[34]. As for possible explanations, Nguyen et al. showed that the three biomarkers praealbumin, platelet factor 4 and S100A12 accurately predict the response of patients with rheumatoid arthritis to TNF inhibitors[35]. Further studies about a gender-specific correlation of these marker could reveal useful findings. In our study, we found that statistically significant less male patients with anti-TNF treatment reported knee/ lower leg and foot/ ankle pain compared to female patients with anti-TNF. This data may indicate that there is a gender difference regarding the effect of anti-TNF therapy for EIM.

One strength of our study is the size of the cohort with 1263 completed questionnaires. Together with our former study evaluating pain in the SIBDCS [3] it is, to the best of our knowledge, the largest evaluation of pain and the use of IBD specific therapy in IBD up to date.

However, our study also has limitations. Due to the study design and the lack of control regarding unreturned questionnaires, a reporting bias cannot be excluded. Patients who actually suffer from pain due to IBD therefore might be overrepresented compared to patients without pain, since the former might be more motivated to return the questionnaire. The patients not responding to the survey might have represented a different phenotype regarding our topic of interest. The existing data of the SIBDCS doesn’t include any information about pain, preventing us from compairing pain specific parameters between responders and non-responders. Furthermore, regarding the use of IBD specific therapy and pain localizations, we do not have information on the reason to initiate medical therapy (i.e. EIM vs. intestinal activity of IBD or both) and how high the prevalence of pain has been before treatment initiation. Our statistical evaluation of the data represents another limitation. We have performed a mostly derscriptive analysis of the dataset. To remain a high response rate and not no overstrain the goodwill of the patients, we intended to keep our questionnaire on a simplistic level. We further see the sizes of our subgroups as a potential limitation. We are aware that small subgroups may be linked to random positive findings. In comparison to other studies about pain in IBD, the subgroups examined here are not considerably small. Furthermore, we aimed to include as many pain localizations as possible to thoroughly analyse the distribution of pain. Additionally, our findings, particularly regarding Anti-TNF, match the clinical observations, depicting a genuine outcome.

In summary, we could show that the frequency of elbow pain was lower in patients treated with anti-TNF but higher under steroid treatment. There were no significant differences regarding the use IBD specific therapy and the character, duration and frequency of pain. Furthermore, our data point towards a higher treatment benefit of anti-TNF with regards to EIM in male patients which should be followed up in future studies.

## Supporting information

S1 Table Pain localization (Steroids). (PDF)

S2 Table Pain localization (5-aminosalicylic acid). (PDF)

S3 Table Pain localization (Immunomodulators). (PDF)

S4 Table Pain localization (Antibiotics). (PDF)

S5 Table Pain localization (Calcineurin-Inhibitors). (PDF)

S6 Table Duration of pain (Steroids). (PDF)

S7 Table Duration of pain (5-aminosalicylic acid). (PDF)

S8 Table Duration of pain (Antibiotics). (PDF)

S9 Table Duration of pain (Calcineurin-Inhibitors). (PDF)

S10 Table Duration of pain (Immunomodulators). (PDF)

S11 Table Frequency of pain (Steroids). (PDF)

S12 Table Frequency of pain (5-aminosalicylic acid). (PDF)

S13 Table Frequency of pain (Antibiotics). (PDF)

S14 Table Frequency of pain (Calcineurin-Inhibitors). (PDF)

S15 Table Frequency of pain (Immunomodulators). (PDF)

S16 Table Pain character (Anti-TNF). (PDF)

S17 Table Pain character (Steroids). (PDF)

S18 Table Pain character (5-aminosalicylic acid). (PDF)

S19 Table Pain character (Antibiotics). (PDF)

S20 Table Pain character (Calcineurin-Inhibitors). (PDF)

S21 Table Pain character (Immunomodulators). (PDF)

S22 Table Duration of pain attacks (Steroids). (PDF)

S23 Table Duration of pain attacks (5-aminosalicylic acid). (PDF)

S24 Table Duration of pain attacks (Antibiotics). (PDF)

S25 Table Duration of pain attacks (Calcineurin-Inhibitors). (PDF)

S26 Table Duration of pain attacks (Immunomodulators). (PDF)
