# Supplementary material for: Association of IBD specific treatment and prevalence of pain in the Swiss IBD cohort study
Source: PLoS One. 2019 Apr 25;14(4):e0215738. doi: 10.1371/journal.pone.0215738 (PMC6483222; doi:10.1371/journal.pone.0215738)
Supplement: S1 Table — (PDF) [file pone.0215738.s001.pdf]

S1 Table: Pain localization (Steroids)

	Steroids	No steroids	
**Pain Localization**	N (%)	N (%)	p-value
**Head**	46 (23.2)	157 (22.6)	0.847
**Neck**	21 (10.6)	102 (14.67)	0.161
**Finger/hand**	44 (22.2)	151 (21.7)	0.922
**Elbow**	28 (14.1)	59 (8.5)	**0.021**
**Shoulder**	43 (21.7)	139 (20)	0.617
**Back**	76 (38.4)	237 (34)	0.272
**Hip/thigh**	47 (23.7)	167 (24)	>0.999
**Knee/lower leg**	61 (30.8)	181 (26)	0.204
**Hock/foot**	33 (16.7)	111 (15.9)	0.826
**Abdomen**	107 (54)	373 (53.6)	0.935
