# Supplementary material for: Association of IBD specific treatment and prevalence of pain in the Swiss IBD cohort study
Source: PLoS One. 2019 Apr 25;14(4):e0215738. doi: 10.1371/journal.pone.0215738 (PMC6483222; doi:10.1371/journal.pone.0215738)
Supplement: S2 Table — (PDF) [file pone.0215738.s002.pdf]

S2 Table: Pain localization (5-aminosalicylic acid)

	**5-aminosalicylic acid**	**No 5-aminosalicylic acid**	
**Pain Localization**	N (%)	N (%)	p-value
**Head**	71 (21.3)	132 (23.6)	0.458
**Neck**	53 (15.9)	70 (12.5)	0.161
**Finger/hand**	69 (20.7)	126 (22.5)	0.558
**Elbow**	30 (9)	57 (10.2)	0.641
**Shoulder**	69 (20.7)	113 (20.2)	0.864
**Back**	108 (32.3)	205 (36.6)	0.218
**Hip/thigh**	84 (25.1)	130 (23.2)	0.517
**Knee/lower leg**	90 (26.9)	152 (27.1)	>0.999
**Hock/foot**	57 (17)	87 (15.5)	>0.999
**Abdomen**	177 (53)	303 (54.1)	0.781
