# Supplementary material for: Association of IBD specific treatment and prevalence of pain in the Swiss IBD cohort study
Source: PLoS One. 2019 Apr 25;14(4):e0215738. doi: 10.1371/journal.pone.0215738 (PMC6483222; doi:10.1371/journal.pone.0215738)
Supplement: S3 Table — (PDF) [file pone.0215738.s003.pdf]

S3 Table: Pain localization (Immunomodulators)

	**Immunomodulators**	**No immunomodulators**	
**Pain Localization**	N (%)	N (%)	p-value
**Head**	67 (21.2)	136 (23.5)	0.453
**Neck**	41 (13)	82 (25.9)	0.684
**Finger/hand**	78 (24.7)	117 (37)	0.128
**Elbow**	31 (9.8)	56 (17.7)	>0.999
**Shoulder**	58 (18.4)	124 (39.2)	0.297
**Back**	112 (35.4)	201 (63.6)	0.883
**Hip/thigh**	78 (24.7)	136 (43)	0.743
**Knee/lower leg**	85 (26.9)	157 (49.7)	>0.999
**Hock/foot**	46 (14.6)	98 (31)	0.392
**Abdomen**	180 (57)	300 (94.3)	0.160
