# Supplementary material for: Association of IBD specific treatment and prevalence of pain in the Swiss IBD cohort study
Source: PLoS One. 2019 Apr 25;14(4):e0215738. doi: 10.1371/journal.pone.0215738 (PMC6483222; doi:10.1371/journal.pone.0215738)
Supplement: S4 Table — (PDF) [file pone.0215738.s004.pdf]

S4 Table: Pain localization (Antibiotics)

	**Antibiotics**	**No antibiotics**	
**Pain Localization**	N (%)	N (%)	p-value
**Head**	2 (18.2)	201 (22.8)	>0.999
**Neck**	2 (18.2)	121 (13.7)	0.654
**Finger/hand**	4 (36.4)	191 (21.6)	0.267
**Elbow**	1 (9)	86 (9.7)	>0.999
**Shoulder**	1 (9.)	181 (20.5)	0.704
**Back**	7 (63.6)	306 (34.7)	0.057
**Hip/thigh**	2 (18.2)	212 (24)	>0.999
**Knee/lower leg**	5 (45.5)	237 (26.8)	0.179
**Hock/foot**	2 (18.2)	142 (16)	0.693
**Abdomen**	4 (36.4)	476 (53.9)	0.362
