# Supplementary material for: Association of IBD specific treatment and prevalence of pain in the Swiss IBD cohort study
Source: PLoS One. 2019 Apr 25;14(4):e0215738. doi: 10.1371/journal.pone.0215738 (PMC6483222; doi:10.1371/journal.pone.0215738)
Supplement: S5 Table — (PDF) [file pone.0215738.s005.pdf]

S5 Table: Pain localization (Calcineurin-Inhibitors)

	**Calcineurin-Inhibitors**	**No calcineurin-inhibitors**	
**Pain Localization**	N (%)	N (%)	p-value
**Head**	3 (25)	200 (22.7)	0.740
**Neck**	3 (25)	120 (13.6)	0.221
**Finger/hand**	2 (16.7)	193 (21.9)	>0.999
**Elbow**	1 (8.3)	86 (9.8)	>0.999
**Shoulder**	2 (16.7)	180 (20.4)	>0.999
**Back**	7 (58.3)	306 (34.7)	0.124
**Hip/thigh**	4 (33.3)	210 (23.8)	0.495
**Knee/lower leg**	1 (8.3)	241 (27.3)	0.197
**Hock/foot**	0 (0)	144 (16.3)	0.231
**Abdomen**	6 (50)	474 (53.7)	>0.999
