# Supplementary material for: Association of IBD specific treatment and prevalence of pain in the Swiss IBD cohort study
Source: PLoS One. 2019 Apr 25;14(4):e0215738. doi: 10.1371/journal.pone.0215738 (PMC6483222; doi:10.1371/journal.pone.0215738)
Supplement: S6 Table — (PDF) [file pone.0215738.s006.pdf]

S6 Table: Duration of pain (Steroids)

	**Steroids**	**No steroids**	
**Pain peroid**	N(%)	N(%)	p-value
**<1 month**	2 (1)	13 (1.9)	0.542
**1 month-½ year**	10 (5)	47 (6.8)	0.509
**½ year-1 year**	12 (6.)	47 (6.8)	0.871
**1-2 years**	14 (7)	65 (9.3)	0.394
**2-5 years**	52 (26.3)	163 (23.4)	0.450
**>5 years**	108 (54.5)	361 (51.9)	0.519
