# Supplementary material for: Association of IBD specific treatment and prevalence of pain in the Swiss IBD cohort study
Source: PLoS One. 2019 Apr 25;14(4):e0215738. doi: 10.1371/journal.pone.0215738 (PMC6483222; doi:10.1371/journal.pone.0215738)
Supplement: S7 Table — (PDF) [file pone.0215738.s007.pdf]

S7 Table: Duration of pain (5-aminosalicylic acid)

	**5-aminosalicylic acid**	**No 5-aminosalicylic acid**	
**Pain peroid**	N(%)	N(%)	p-value
**<1 month**	6 (1.8)	9 (1.6)	0.795
**1 month-½ year**	18 (5.4)	39 (7)	0.397
**½ year-1 year**	24 (7.2)	35 (6.3)	0.580
**1-2 years**	35 (10.5)	44 (7.9)	0.182
**2-5 years**	89 (26.6)	126 (22.5)	0.169
**>5 years**	162 (48.5)	307 (54.8)	0.072
