# Supplementary material for: Association of IBD specific treatment and prevalence of pain in the Swiss IBD cohort study
Source: PLoS One. 2019 Apr 25;14(4):e0215738. doi: 10.1371/journal.pone.0215738 (PMC6483222; doi:10.1371/journal.pone.0215738)
Supplement: S8 Table — (PDF) [file pone.0215738.s008.pdf]

S8 Table: Duration of pain (Antibiotics)

	Antibiotics	No antibiotics	
**Pain peroid**	N(%)	N(%)	p-value
**<1 month**	0 (0)	15 (1.7)	>0.999
**1 month-½ year**	1 (9.1)	56 (6.3)	0.517
**½ year-1 year**	0 (0)	59 (6.7)	>0.999
**1-2 years**	0 (0)	79 (8.9)	0.612
**2-5 years**	2 (18.2)	213 (24.1)	>0.999
**>5 years**	8 (72.7)	461 (52.2)	0.229
