# Supplementary material for: Association of IBD specific treatment and prevalence of pain in the Swiss IBD cohort study
Source: PLoS One. 2019 Apr 25;14(4):e0215738. doi: 10.1371/journal.pone.0215738 (PMC6483222; doi:10.1371/journal.pone.0215738)
Supplement: S9 Table — (PDF) [file pone.0215738.s009.pdf]

S9 Table: Duration of pain (Calcineurin-Inhibitors)

	Calcineurin-Inhibitors	No calcineurin-inhibitors	
**Pain peroid**	N(%)	N(%)	p-value
**<1 month**	1 (8.3)	14 (1.6)	0.184
**1 month-½ year**	1 (8.3)	56 (6.3)	0.548
**½ year-1 year**	1 (8.3)	58 (6.6)	0.561
**1-2 years**	1 (8.3)	78 (8.8)	>0.999
**2-5 years**	2 (16.7)	213 (24.1)	0.740
**>5 years**	6 (50)	463 (52.5)	>0.999
