# Supplementary material for: Association of IBD specific treatment and prevalence of pain in the Swiss IBD cohort study
Source: PLoS One. 2019 Apr 25;14(4):e0215738. doi: 10.1371/journal.pone.0215738 (PMC6483222; doi:10.1371/journal.pone.0215738)
Supplement: S10 Table — (PDF) [file pone.0215738.s010.pdf]

S10 Table: Duration of pain (Immunomodulators)

	Immunomodulators	No immunomodulators	
**Pain peroid**	N(%)	N(%)	p-value
**<1 month**	6 (1.9)	9 (1.6)	0.786
**1 month-½ year**	24 (7.6)	33 (5.7)	0.316
**½ year-1 year**	23 (7.3)	36 (6.2)	0.574
**1-2 years**	22 (7)	57 (9.9)	0.174
**2-5 years**	78 (24.7)	137 (23.8)	0.744
**>5 years**	136 (51.6)	306 (52.9)	0.726
