# Supplementary material for: Association of IBD specific treatment and prevalence of pain in the Swiss IBD cohort study
Source: PLoS One. 2019 Apr 25;14(4):e0215738. doi: 10.1371/journal.pone.0215738 (PMC6483222; doi:10.1371/journal.pone.0215738)
Supplement: S11 Table — (PDF) [file pone.0215738.s011.pdf]

S11 Table: Frequency of pain (Steroids)

	**Steroids**	**No steroids**	
**Pain Frequency**	N(%)	N(%)	p-value
**Several times daily**	35 (22.3)	129 (23.9)	0.748
**1x/day**	6 (3.8)	39 (7.2)	0.142
**Several times per week**	37 (23.6)	97 (18)	0.134
**1/week**	12 (7.6)	25 (4.6)	0.156
**Several times per month**	32 (20.4)	98 (18.1)	0.560
**1x/month**	12 (7.6)	55 (10.2)	0.441
**<1x/month**	23 (14.6)	97 (18)	0.400
