# Supplementary material for: Association of IBD specific treatment and prevalence of pain in the Swiss IBD cohort study
Source: PLoS One. 2019 Apr 25;14(4):e0215738. doi: 10.1371/journal.pone.0215738 (PMC6483222; doi:10.1371/journal.pone.0215738)
Supplement: S12 Table — (PDF) [file pone.0215738.s012.pdf]

S12 Table: Frequency of pain (5-aminosalicylic acid)

	5-aminosalicylic acid	No 5-aminosalicylic acid	
**Pain Frequency**	N(%)	N(%)	p-value
**Several times daily**	53 (21.3)	111 (5.8)	0.307
**1x/day**	19 (7.6)	26 (5.8)	0.340
**Several times per week**	53 (21.3)	81 (18.1)	0.316
**1/week**	15 (6)	22 (4.9)	0.597
**Several times per month**	40 (16.1)	90 (20.1)	0.223
**1x/month**	27 (10.8)	40 (8.9)	0.423
**<1x/month**	42 (16.9)	78 (17.4)	0.916
