# Supplementary material for: Association of IBD specific treatment and prevalence of pain in the Swiss IBD cohort study
Source: PLoS One. 2019 Apr 25;14(4):e0215738. doi: 10.1371/journal.pone.0215738 (PMC6483222; doi:10.1371/journal.pone.0215738)
Supplement: S13 Table — (PDF) [file pone.0215738.s013.pdf]

S13 Table: Frequency of pain (Antibiotics)

	Antibiotics	No antibiotics	
**Pain Frequency**	N(%)	N(%)	p-value
**Several times daily**	2 (20)	162 (23.6)	>0.999
**1x/day**	0 (0)	45 (6.6)	>0.999
**Several times per week**	2 (20)	132 (19.2)	>0.999
**1/week**	1 (10)	36 (5.2)	0.422
**Several times per month**	1 (10)	129 (18.8)	0.697
**1x/month**	0 (0)	67 (9.8)	0.610
**<1x/month**	4 (40)	116 (16.9)	0.075
