# Supplementary material for: Association of IBD specific treatment and prevalence of pain in the Swiss IBD cohort study
Source: PLoS One. 2019 Apr 25;14(4):e0215738. doi: 10.1371/journal.pone.0215738 (PMC6483222; doi:10.1371/journal.pone.0215738)
Supplement: S14 Table — (PDF) [file pone.0215738.s014.pdf]

## S14 Table: Frequency of pain (Calcineurin-Inhibitors)

| | **Calcineurin-Inhibitors** | **No calcineurin-inhibitors** | |
|---|---|---|---|
| **Pain Frequency** | N(%) | N(%) | p-value |
| **Several times daily** | 4 (20) | 160 (23.2) | 0.093 |
| **1x/day** | 0 (0) | 45 (6.5) | >0.999 |
| **Several times per week** | 3 (20) | 131 (19) | 0.185 |
| **1/week** | 0 (10) | 37 (5.4) | >0.999 |
| **Several times per month** | 0 (10) | 130 (18.9) | 0.363 |
| **1x/month** | 1 (0) | 66 (9.6) | 0.556 |
| **<1x/month** | 0 (40) | 120 (17.4) | 0.362 |
