# Supplementary material for: Association of IBD specific treatment and prevalence of pain in the Swiss IBD cohort study
Source: PLoS One. 2019 Apr 25;14(4):e0215738. doi: 10.1371/journal.pone.0215738 (PMC6483222; doi:10.1371/journal.pone.0215738)
Supplement: S15 Table — (PDF) [file pone.0215738.s015.pdf]

S15 Table: Frequency of pain (Immunomodulators)

	Immunomodulators	No immunomodulators	
**Pain Frequency**	N(%)	N(%)	p-value
**Several times daily**	65 (25.5)	99 (22.4)	0.355
**1x/day**	19 (7.5)	26 (5.9)	0.427
**Several times per week**	52 (20.4)	82 (18.6)	0.551
**1/week**	11 (4.3)	26 (5.9)	0.483
**Several times per month**	46 (18)	84 (19)	0.840
**1x/month**	26 (10.2)	41 (9.3)	0.690
**<1x/month**	36 (14.1)	84 (19)	0.118
