# Supplementary material for: Association of IBD specific treatment and prevalence of pain in the Swiss IBD cohort study
Source: PLoS One. 2019 Apr 25;14(4):e0215738. doi: 10.1371/journal.pone.0215738 (PMC6483222; doi:10.1371/journal.pone.0215738)
Supplement: S16 Table — (PDF) [file pone.0215738.s016.pdf]

S16 Table: Pain character (Anti-TNF)

	**Anti-TNF**	**No anti-TNF**	
**Pain Charakter**	N (%)	N (%)	p-value
**Constant pain w/ slight fluctuation**	36 (19)	115 (19)	>0.999
**Constant pain w/ strong fluctuation**	15 (7.9)	64 (10.6)	0.331
**Pain attacks w/ pain free intervals**	115 (60.5)	349 (57.9)	0.554
**Pain attacks w/ constant pain**	30 (15.8)	75 (12.4)	0.268
