# Supplementary material for: Association of IBD specific treatment and prevalence of pain in the Swiss IBD cohort study
Source: PLoS One. 2019 Apr 25;14(4):e0215738. doi: 10.1371/journal.pone.0215738 (PMC6483222; doi:10.1371/journal.pone.0215738)
Supplement: S17 Table — (PDF) [file pone.0215738.s017.pdf]

S17 Table: Pain character (Steroids)

	Steroids	No steroids	
**Pain Charakter**	N (%)	N (%)	p-value
**Constant pain w/ slight fluctuation**	37 (20.2)	114 (18.5)	0.592
**Constant pain w/ strong fluctuation**	15 (8.2)	64 (10.4)	0.480
**Pain attacks w/ pain free intervals**	109 (59.6)	355 (57.6)	0.670
**Pain attacks w/ constant pain**	22 (12)	83 (13.5)	0.708
