# Supplementary material for: Association of IBD specific treatment and prevalence of pain in the Swiss IBD cohort study
Source: PLoS One. 2019 Apr 25;14(4):e0215738. doi: 10.1371/journal.pone.0215738 (PMC6483222; doi:10.1371/journal.pone.0215738)
Supplement: S18 Table — (PDF) [file pone.0215738.s018.pdf]

S18 Table: Pain character (5-aminosalicylic acid)

	**5-aminosalicylic acid**	**No 5-aminosalicylic acid**	
**Pain Charakter**	N (%)	N (%)	p-value
**Constant pain w/ slight fluctuation**	56 (19.3)	95 (18.7)	0.851
**Constant pain w/ strong fluctuation**	33 (11.4)	46 (9)	0.324
**Pain attacks w/ pain free intervals**	171 (59)	293 (57.6)	0.709
**Pain attacks w/ constant pain**	30 (10.3)	75 (14.7)	0.082
