# Supplementary material for: Association of IBD specific treatment and prevalence of pain in the Swiss IBD cohort study
Source: PLoS One. 2019 Apr 25;14(4):e0215738. doi: 10.1371/journal.pone.0215738 (PMC6483222; doi:10.1371/journal.pone.0215738)
Supplement: S19 Table — (PDF) [file pone.0215738.s019.pdf]

S19 Table: Pain character (Antibiotics)

	Antibiotics	No antibiotics	
**Pain Charakter**	N (%)	N (%)	p-value
**Constant pain w/ slight fluctuation**	1 (11.1)	150 (19)	>0.999
**Constant pain w/ strong fluctuation**	1 (11.1)	78 (9.9)	0.610
**Pain attacks w/ pain free intervals**	7 (77.8)	457 (57.8)	0.316
**Pain attacks w/ constant pain**	0 (0)	105 (13.3)	0.615
