# Supplementary material for: Association of IBD specific treatment and prevalence of pain in the Swiss IBD cohort study
Source: PLoS One. 2019 Apr 25;14(4):e0215738. doi: 10.1371/journal.pone.0215738 (PMC6483222; doi:10.1371/journal.pone.0215738)
Supplement: S20 Table — (PDF) [file pone.0215738.s020.pdf]

S20 Table: Pain character (Calcineurin-Inhibitors)

	Calcineurin-Inhibitors	No calcineurin-inhibitors	
**Pain Charakter**	N (%)	N (%)	p-value
**Constant pain w/ slight fluctuation**	4 (36.4)	147 (18.7)	0.136
**Constant pain w/ strong fluctuation**	2 (18.2)	77 (9.8)	0.297
**Pain attacks w/ pain free intervals**	4 (36.4)	460 (58.4)	0.217
**Pain attacks w/ constant pain**	1 (9.1)	104 (13.2)	>0.999
