# Supplementary material for: Association of IBD specific treatment and prevalence of pain in the Swiss IBD cohort study
Source: PLoS One. 2019 Apr 25;14(4):e0215738. doi: 10.1371/journal.pone.0215738 (PMC6483222; doi:10.1371/journal.pone.0215738)
Supplement: S21 Table — (PDF) [file pone.0215738.s021.pdf]

S21 Table: Pain character (Immunomodulators)

	Immunomodulators	No immunomodulators	
**Pain Charakter**	N (%)	N (%)	p-value
**Constant pain w/ slight fluctuation**	49 (17.3)	102 (19.8)	0.396
**Constant pain w/ strong fluctuation**	31 (11)	48 (9.3)	0.460
**Pain attacks w/ pain free intervals**	164 (57.7)	300 (58.3)	0.940
**Pain attacks w/ constant pain**	40 (14.1)	65 (12.6)	0.585
