# Supplementary material for: Association of IBD specific treatment and prevalence of pain in the Swiss IBD cohort study
Source: PLoS One. 2019 Apr 25;14(4):e0215738. doi: 10.1371/journal.pone.0215738 (PMC6483222; doi:10.1371/journal.pone.0215738)
Supplement: S22 Table — (PDF) [file pone.0215738.s022.pdf]

S22 Table: Duration of pain attacks (Steroids)

	**Steroids**	**No steroids**	
**Pain Attacks**	N (%)	N (%)	p-value
**Seconds**	15 (9.8)	72 (13.4)	0.270
**Minutes**	41 (26.8)	173 (32.3)	0.234
**Hours**	54 (35.3)	176 (32.8)	0.561
**<3 days**	26 (17)	62 (11.6)	0.098
**>5 days**	17 (11.1)	53 (9.9)	0.650
