# Supplementary material for: Association of IBD specific treatment and prevalence of pain in the Swiss IBD cohort study
Source: PLoS One. 2019 Apr 25;14(4):e0215738. doi: 10.1371/journal.pone.0215738 (PMC6483222; doi:10.1371/journal.pone.0215738)
Supplement: S23 Table — (PDF) [file pone.0215738.s023.pdf]

S23 Table: Duration of pain attacks (5-aminosalicylic acid)

	5-aminosalicylic acid	No 5-aminosalicylic acid	
**Pain Attacks**	N (%)	N (%)	p-value
**Seconds**	35 (14.1)	52 (11.8)	0.403
**Minutes**	70 (28.2)	144 (32.7)	0.264
**Hours**	87 (35.1)	143 (32.4)	0.501
**<3 days**	28 (11.3)	60 (13.6)	0.404
**>5 days**	28 (11.3)	42 (9.5)	0.511
