# Supplementary material for: Association of IBD specific treatment and prevalence of pain in the Swiss IBD cohort study
Source: PLoS One. 2019 Apr 25;14(4):e0215738. doi: 10.1371/journal.pone.0215738 (PMC6483222; doi:10.1371/journal.pone.0215738)
Supplement: S24 Table — (PDF) [file pone.0215738.s024.pdf]

S24 Table: Duration of pain attacks (Antibiotics)

	**Antibiotics**	**No antibiotics**	
**Pain Attacks**	N (%)	N (%)	p-value
**Seconds**	2 (20)	85 (12.5)	0.366
**Minutes**	1 (10)	213 (31.4)	0.185
**Hours**	4 (40)	226 (33.3)	0.738
**<3 days**	2 (20)	86 (12.7)	0.372
**>5 days**	1 (10)	69 (10.2)	>0.999
