# Supplementary material for: Association of IBD specific treatment and prevalence of pain in the Swiss IBD cohort study
Source: PLoS One. 2019 Apr 25;14(4):e0215738. doi: 10.1371/journal.pone.0215738 (PMC6483222; doi:10.1371/journal.pone.0215738)
Supplement: S25 Table — (PDF) [file pone.0215738.s025.pdf]

S25 Table: Duration of pain attacks (Calcineurin-Inhibitors)

	**Calcineurin-Inhibitors**	**No calcineurin-inhibitors**	
**Pain Attacks**	N (%)	N (%)	p-value
**Seconds**	0 (0)	87 (12.8)	0.605
**Minutes**	4 (50)	210 (30.8)	0.262
**Hours**	2 (25)	228 (33.5)	0.725
**<3 days**	1 (12.5)	87 (12.8)	>0.999
**>5 days**	1 (12.5)	69 (10.1)	0.577
