# Supplementary material for: Association of IBD specific treatment and prevalence of pain in the Swiss IBD cohort study
Source: PLoS One. 2019 Apr 25;14(4):e0215738. doi: 10.1371/journal.pone.0215738 (PMC6483222; doi:10.1371/journal.pone.0215738)
Supplement: S26 Table — (PDF) [file pone.0215738.s026.pdf]

S26 Table: Duration of pain attacks (Immunomodulators)

	**Immunomodulators**	**No immunomodulators**	
**Pain Attacks**	N (%)	N (%)	p-value
**Seconds**	34 (13.6)	53 (12.1)	0.553
**Minutes**	78 (31.2)	136 (31)	>0.999
**Hours**	84 (33.6)	146 (33.3)	0.933
**<3 days**	28 (11.2)	60 (13.7)	0.406
**>5 days**	26 (10.4)	44 (10)	0.896
